# Negative Correlation between Altitude and COVID-19 Pandemic in Colombia: A Preliminary Report

**DOI:** 10.4269/ajtmh.20-1027

**Published:** 2020-10-26

**Authors:** Eder Cano-Pérez, Jaison Torres-Pacheco, María Carolina Fragozo-Ramos, Génesis García-Díaz, Eduardo Montalvo-Varela, Juan Carlos Pozo-Palacios

**Affiliations:** 1 Molecular Research Unit Group (UNIMOL), Laboratory of Tropical Medicine, University of Cartagena, Cartagena de Indias, Colombia;; 2 Department of Internal Medicine, Faculty of Medicine, University of Cartagena, Cartagena de Indias, Colombia;; 3 Centro Especializado en Genética Médica (CEGEMED), Quito, Ecuador;; 4 Department of Clinical Biochemistry, Faculty of Chemical Sciences, Central University of Ecuador, Quito, Ecuador;; 5 Department of Epidemiology, Faculty of Medical Science, University of Cuenca, Cuenca, Ecuador

## Abstract

It has been suggested that high altitude can reduce the infectivity and case fatality rate of COVID-19. We investigated the relationship between altitude and the COVID-19 pandemic in Colombia. Epidemiological data included the number of positive cases, deaths, and the case fatality rate of COVID-19. In particular, we analyzed data from 70 cities with altitudes between 1 and 3,180 m. Correlations and linear regression models adjusted to population density were performed to examine the relationship and contribution of altitude to epidemiological variables. The case fatality rate was negatively correlated with the altitude of the cities. The incidence of cases and deaths from COVID-19 had an apparent correlation with altitude; however, these variables were better explained by population density. In general, these findings suggest that living at high altitude can reduce the impact of COVID-19, especially the case fatality rate.

Since its appearance in December 2019 in Wuhan, China, the SARS-CoV-2 virus, causative agent of COVID-19, has spread throughout the world, infecting at least 20 million people and causing approximately 745,000 deaths.^1^ SARS-CoV-2 uses angiotensin-converting enzyme 2 (ACE2) receptors to enter human cells.^2^ Angiotensin-converting enzyme 2 is part of the renin–angiotensin–aldosterone system, present in several organs; this enzyme opposes the functions of angiotensin (Ang) II by converting Ang II into Ang (1–7), which has vasodilator effects unlike the product of its counterpart, the homologous enzyme ACE1, which has powerful vasoconstrictive properties.^3^ Angiotensin-converting enzyme 2 receptors are widely expressed in the pulmonary alveolar epithelium, making these organs the main targets for SARS-CoV-2 infection and facilitating the development of complications such as pneumonia, acute respiratory distress syndrome, and death.^4^ Therefore, these receptors play a vital role in the progression and prognosis of the disease.

A decrease in the expression of ACE2 has been reported in conditions of chronic hypoxia in different experimental models,^5,6^ which allows speculating whether hypobaric hypoxia at high altitude reduces the tissue expression of ACE2 that could protect against SARS-CoV-2 virus in people living at high altitudes; however, the current evidence on decreased ACE2 expression in hypoxia may not be sufficient to support this hypothesis.^7^ Recently, an epidemiological study described a lower incidence of COVID-19 in high-altitude areas; the authors raised a possible weaker transmission rate of SARS-CoV-2 and less severity in these populations. The proposed protective mechanisms are based on the compromised half-life of the virus and hypoxia-mediated downregulated ACE2 expression that is present in high-altitude environments.^8^ However, other studies reported that elevated altitude does not induce a decrease in the COVID-19 case fatality rate.^9,10^

Considering this background, we decided to examine the scenario of positive cases, deaths, and the case fatality rate of COVID-19 in Colombia in an altitude range from 1 to 3,180 m above sea level. Data from 70 municipalities affected by the pandemic were used, including the 32 departmental capital cities of the country. Data on positive cases and deaths by COVID-19 were obtained from the open files of the Colombian Ministry of Health from August 1, 2020.^11^ Population and altitude information was obtained from the Administrative Department of National Statistics and reference geographic institution in Colombia, respectively.^12,13^ The case fatality rates for each city were evaluated; the formula used was: number of deaths from COVID-19 among incident cases divided by the total number of incident cases ×100.

As data were not normally distributed, Spearman’s rank correlation test was performed to examine the relationship among altitude and positives cases, deaths for COVID-19, and the case fatality rate. A multiple linear regression model was performed incorporating the population density of the cities to assess the contribution of altitude to the explanation power of the models for epidemiological variables. First, the adjusted *R*^2^ values of the linear regression models were measured while accounting for only the population density of the cities. Next, altitude was added to the linear regression analysis, and new adjusted *R*^2^ values were calculated. Statistical analyses were performed using the IBM (IBM Corp, Armonk, NY), SPSS statistical software version 19.0. Statistical significance was defined as *P* < 0.05.

As of August 1, 306,181 positive cases and 10,822 deaths had been reported in Colombia, representing a cumulative case fatality rate of 3.5%. The cities with the highest positive cases were Bogotá (2,567 m) with 104,656, Barranquilla (18 m) with 30,062, and Cali (1,004 m) with 19,691 positives cases. Likewise, they were the cities with the most deaths from COVID-19. These data alone do not suggest any trend between altitude and COVID-19 cases and deaths.

We observe that in Bogotá (2,567 m), the case fatality rate was 2.8%. Other high-altitude Andean cities such as Tunja (2,690 m) and Pasto (2,871) showed similar values, presenting 2.6% and 2.6% in the case fatality rate, respectively. When evaluating coastal cities or with altitudes below 200 m, we found relatively higher case fatality rates, the most notable examples being Cereté (12 m), Inírida (100 m), and Montería (36 m) with case fatality rates of 15.6%, 11.1%, and 10.6%, respectively. Statistical analyses found a negative correlation between the altitude of cities and the COVID-19 case fatality rate. In Table 1, a correlation coefficient of −0.451 was observed with a *P*-value < 0.01. Altitude continued to be an influential factor in the case fatality rate in the multiple regression analysis that incorporated the population density of cities. The adjusted *R*^2^ value of the regression model that included only the population density was −0.013; the addition of altitude in the model improved the value of *R*^2^ to 0.118. Simple correlation coefficients between altitude and positive cases and deaths for COVID-19 were similar to the case fatality rate; however, linear regression models showed that these variables are better explained by population density.

**Table 1:** Correlation coefficients and adjusted *R*^2^ values among altitude and positive cases, deaths for COVID-19, and case fatality rate

Statistical test	Total positive cases	Total deaths	Case fatality rate
Correlation coefficient	−0.315*	−0.396*	−0.451*
Adjusted *R*^2^ of the regression models that included population density	0.092*	0.121*	−0.013
Adjusted *R*^2^ of the regression models that included population density and altitude	0.082†	0.108*	0.118*

* Correlation is significant at level 0.01.

† Correlation is significant at level 0.05.

In the present study, the relationship between altitude and the COVID-19 pandemic in Colombia in a height range between 2 and 3,180 m was analyzed. Our findings suggest that high altitudes can induce a decrease in the COVID-19 case fatality rate. These results are in line with other studies that suggest that high altitudes can protect against the pathogenesis and severity of SARS-COV-2 infection.^8,14^ By contrast, our results differ from those obtained by Woolcott and Bergman in a recent study conducted in the United States and Mexico, which reports a higher mortality rate at high altitude for both countries.^9^ Similarly, Segovia-Juarez et al.,^10^ in Peru, found that high altitudes decrease the COVID-19 infection rate but not the case fatality rate. Differences in results are likely to be related to the number of cities analyzed. In the Peru study, 185 provincial capitals in altitude ranges between 3 and 4,342 m were analyzed; in the U.S. and Mexico study, 1,016 counties and 567 municipalities were analyzed, respectively. Also, other intrinsic factors such as ethnic and genetic differences in populations, host comorbidities, medical care system, environmental factors related to viral transmission, social structure, and the extent of isolation measures in each region may influence the epidemiological dynamics of COVID-19.^7^

Population and/or population density are known to be common factors for the spread of infectious diseases, and, as expected, have been reported as one of the main components in the amplification of the COVID-19 pandemic.^15,16^ In this study, we found that positive cases and deaths for COVID-19 had an apparent correlation with altitude; however, these variables were better explained by population density; this trend can be explained by understanding that the larger the population is, the higher the transportation volumes, economic activity, air pollution, social interactions, and virus infections are.

The results of this preliminary study show a negative correlation between altitude and the impact of the COVID-19 pandemic in Colombia, especially with the case fatality rate of disease. The current study is exploratory and offers some limitations. First, a large proportion of municipalities of the country were not included. Also, other social, climatic, environmental, and clinical variables were not estimated. Therefore, additional studies are required to corroborate the data presented here.
